# Cutaneous Leishmaniasis Caused by an Unknown *Leishmania* Strain, Arizona, USA

**DOI:** 10.3201/eid2706.204198

**Published:** 2021-06

**Authors:** Marcos de Almeida, Yueli Zheng, Fernanda S. Nascimento, Henry Bishop, Vitaliano A. Cama, Dhwani Batra, Yvette Unoarumhi, Abaseen K. Afghan, Vivian Y. Shi, Philip E. LeBoit, Eugene W. Liu, Fariba M. Donovan

**Affiliations:** Centers for Disease Control and Prevention, Atlanta, Georgia, USA (M. de Almeida, Y. Zheng, F.S. Nascimento, H. Bishop, V.A. Cama, D. Batra, Y. Unoarumhi, E.W. Liu);; Eagle Global Scientific, San Antonio, Texas, USA (Y. Zheng);; University of Arizona College of Medicine, Tucson, Arizona, USA (A.K. Afghan, V.Y. Shi, F.M. Donovan);; University of California, San Francisco, California, USA (P.E. LeBoit)

**Keywords:** cutaneous leishmaniasis, autochthonous leishmaniasis, leishmaniasis, Leishmania, parasites, unknown strain, subgenus, clinical case, vector-borne infections, zoonoses, Arizona, United States

## Abstract

We investigated an autochthonous case of cutaneous leishmaniasis caused by a genetically different *Leishmania* sp. in a patient in Arizona, USA. This parasite was classified into the subgenus *Leishmania* on the basis of multilocus DNA sequence and phylogenetic analyses of the rRNA locus and 11 reference genes.

Human leishmaniasis is a vectorborne disease occurring mostly in Central and South America, the Europe/Africa Mediterranean area, the Middle East, and the Indian subcontinent. This disease is caused by parasites in the *Leishmania* subgenera *Viannia* and *Leishmania*, affects ≈2.5 million persons, and causes 60,000 deaths yearly worldwide (*1*). The disease has 3 main clinical forms: cutaneous leishmaniasis (CL), the most prevalent form and caused by species in both *Viannia* and *Leishmania* subgenera; mucocutaneous leishmaniasis, caused by species in the subgenus *Viannia*; and visceral leishmaniasis, caused by *L*. (*L*.) *donovani* and *L*. (*L*.) *infantum*. These syndromes might lead to social stigma because of permanent scars, skin disfigurement, and partial/total destruction of oral/nasopharyngeal mucosa and can result in systemic symptoms including splenomegaly, wasting, and even death (*2*).

Species-specific *Leishmania* identification is critical in clinical management and epidemiologic investigations (*2*). Detection and identification of *Leishmania* parasites were traditionally done through microscopic and multilocus enzyme electrophoresis analysis. Currently, PCR-based methods and multilocus DNA sequence analyses (MLSA) combined with next-generation sequencing, have improved phylogenetic resolution and provided insights into parasite identification, classification, genetic polymorphism, virulence, and drug resistance (*3*,*4*).

*Leishmania* parasites are emerging in previously nonendemic areas (*5*); traditional and exotic *Leishmania* species/strains have been reported in focal areas of the Americas, Europe, Africa, Asia, and the Western Pacific (*6*). In the United States, leishmaniasis is mostly nonreportable and historically considered a travel-associated disease. However, the activity of natural vectors of *Leishmania* and occurrence of autochthonous zoonotic cases of CL and visceral leishmaniasis caused by *L*. (*L*.) *mexicana* or *L*. (*L*.) *infantum* have been reported in several states, including Alabama, Arizona, Arkansas, Delaware, Georgia, Kentucky, Louisiana, Maryland, Mississippi, Ohio, Oklahoma, South Carolina, and Texas (*7*–*10*). Those reports suggest the possibility of local transmission of leishmaniasis, especially in the southwestern US region. We report an autochthonous case of CL from Arizona, USA, caused by an unknown parasite in the subgenus *Leishmania*.

## The Study

In December 2017, a 72-year-old woman from Pima County, Arizona, sought medical care for 2 discrete, progressive, edematous, violaceous papular lesions on the low back. The patient had a history of granulomatosis with polyangiitis, chronic sinusitis, chronic kidney disease, and pulmonary coccidioidomycosis. The patient had never traveled internationally and did not have an underlying health condition predisposing her to leishmaniasis.

In January 2018, after a third lesion erupted, we performed skin biopsies. Histologic sections showed nonnecrotizing granulomas in the papillary dermis, and tiny, basophilic, spherical inclusions within histiocyte cytoplasm resembling amastigotes, suggestive of CL. The lesions showed a limited extent and spontaneous improvement; therefore, no specific treatment was prescribed. The patient was followed for almost 2 years, and the 3 skin lesions remained nodular without ulceration, which eventually resolved by December 2019. The patient remained otherwise asymptomatic.

**Figure 1:**
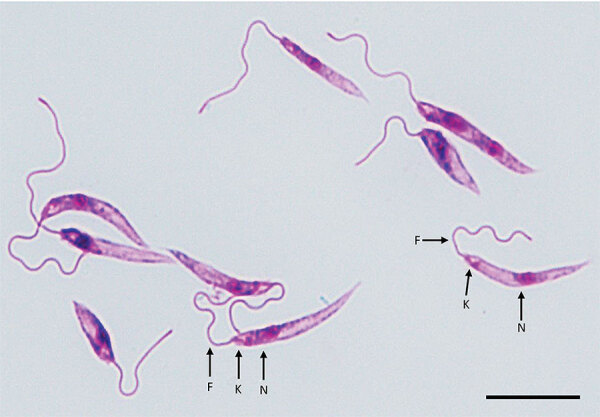
Cultured *Leishmania* promastigotes of the strain isolated from a patient in Arizona, USA. Morphologic features include a slender elongated body that contains a kinetoplast (K) anterior to the nucleus (N) and flagellum (F). The parasite had a total body length of ≈15 μm. Giemsa-stained; scale bar indicates 10 μm.

We tested clinical specimens from the patient following the Centers for Disease Control and Prevention (CDC)–approved protocol for using residual specimens from human subjects (use of residual diagnostic specimens from humans for laboratory methods research protocol no. 6756). We used lesion biopsy specimens submitted to CDC for DNA extraction, touch-prep smears, and in vitro culture in Roswell Park Memorial Institute medium (GIBCO-Thermo-Fisher, https://www.thermofisher.com) containing 15% fetal bovine serum at 25°C (*11*). Microscopic analysis of touch-prep smears identified a large number of amastigotes, whereas promastigotes with cellular shape and architecture compatible with species in the subgenus *Leishmania* were observed from culture (Figure 1).

We extracted DNA from biopsy specimens, cultured parasites by using the DNeasy Blood and Tissue Kit (QIAGEN, https://www.qiagen.com), and amplified the internal transcribed spacer 2 (ITS2) locus by using PCR. We then Sanger sequenced amplicons bidirectionally, assembled by using Lasergene Seqman Pro Software (DNASTAR, Inc., https://www.dnastar.com), and compared with sequences in the GenBank database by using BLASTn (https://blast.ncbi.nlm.nih.gov/Blast.cgi) (*11*).

The resulting sequence (380 bp) (GenBank accession no. MT764332) had low similarity with *L*. (*L*.) *donovani* HQ830358 (90.36%), *L*. (*L*.) *infantum* AJ 634370 (89.9%), *Leishmania* sp. FM209179 (89.5%), *L*. (*L*.) *tropica* FJ948457 (89.2%), *L*. (*L*.) *mexicana* FJ948437 (85.1%), and species in the subgenus *Viannia* (<80.0%). We also tested DNA samples for amplicon melting temperature by using a SYBR green real-time, quantitative PCR protocol, which enables presumptive discrimination of *Leishmania* species (*12*). This analysis showed a melting temperature of 79.5°C, indicating *L*. (*L*.) *infantum* infection. On the basis of PCR analysis, the case-patient was identified as being infected with a *Leishmania* spp., without providing species-level identification.

We used DNA extracted from cultured parasites by using the MagAttract HMW DNA Kit (QIAGEN) to prepare genomic libraries by using the NEBNext Ultra II DNA Library Prep (New England Biolabs, https://www.neb.com) and subjected them to whole-genome sequencing by using the MiSeq platform (Illumina, https://www.illumina.com). MiSeq sequencing resulted in 22,808,630 *Leishmania* reads that had >100× coverage, 3,464 contigs of 29,491,421 bp, and a GC content of 59.68%.

**Figure 2:**
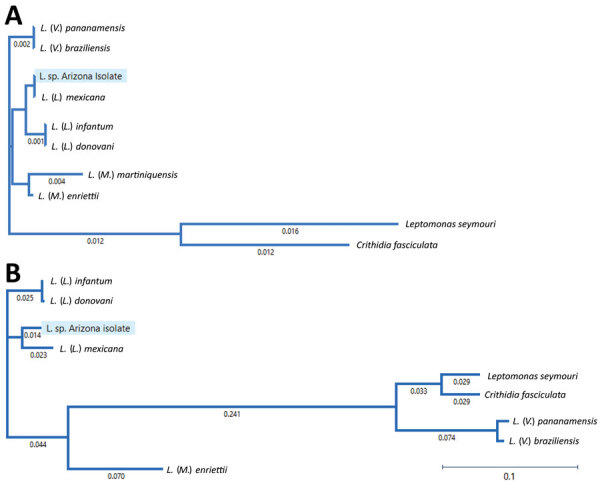
Phylogenetic tree of *Leishmania* subgenus isolates from a patient in Arizona, USA, and reference *Leishmania* species in relationship to species in the subgenera *Leishmania*, *Viannia*, and *Mundina*. A) Phylogenetic tree of *Leishmania* 18S rRNA genes. Sequences of *Crithidia fasciculata* and *Leptomonas seymouri* are included as references. *L*. (*V*.) *panamensis* (GenBank accession no. GQ332362); *L*. (*V*.) *braziliensis* (accession no. GQ332355); *L*. (*L*) *mexinana* (accession no. GQ332260); *L*. (*L*.) *infantum* (accession no. GQ332359); *L*. (*L*.) *donovani* (accession no. GQ332356); *L*. (*M*.) *martiniquensis* (accession no. AF303938); *L*. (*M*.) *enriettii* (accession no. ATAF02000704); *Leptomonas seymore* (accession no. KP717894); and *Crithidia fasciculata* (accession no. Y00055). The 2 non-*Leishmania* trypanosomatids (*Leptomonas seymore* and *Crithidia fasciculata*) were included in the phylogenetic tree because they were previously described as co-infecting parasites in human leishmaniasis cases. B) Phylogenetic tree of glyceraldehyde-3-phosphate dehydrogenase genes. Sequences from *Crithidia fasciculata* and *Leptomonas seymouri* were included as references. Numbers along branches indicate bootstrap values. Scale bars indicate nucleotide substitutions per site.

We conducted MLSA by comparing open reading frames (ORFs) of MiSeq data against GenBank reference sequences at the following loci: β-actin, aspartate aminotransferase, cytosolic glyceraldehyde-3-phosphate dehydrogenase, glucose-6-phosphate dehydrogenase, glucose-6-phosphate isomerase, isocitrate dehydrogenase, cytosolic nicotinamide adenine dinucleotide phosphate, malic enzyme, mannose phosphate isomerase, 6-phosphogluconate dehydrogenase, 6-phosphoglucomutase, heat-shock protein 70, 18SrRNA ITS region and rRNA. We determined similarities between MiSeq and database ORFs, fragment length, and GenBank accession no. (Table). Similarities to *Viannia* reference sequences were 99.6% for 18S rRNA and 65.23% for ITS rRNA loci. To visualize the taxonomic location of the isolate from Arizona, we constructed an evolutionary distance tree by using MiSeq 18SrRNA and cytosolic glyceraldehyde-3-phosphate dehydrogenase ORFs, as well as complete reference sequences in the subgenera *Leishmania*, *Viannia*, and *Mundina* (Figure 2).

**Table:** Comparative analysis of sequences of 13 genes showing percent sequence identity between a *Leishmania* isolate from a patient in Arizona, USA, and other *Leishmania* species*

Gene ORF†	Arizona isolate *vs. L.* (*L.*) *donovani*			Arizona isolate *vs. L.* (*L*.) *infantum*			Arizona isolate *vs. L.* (*L*.) *mexicana*			Arizona isolate *vs. L.* (*M.*) *enrietti*			Arizona isolate *vs. L.* (*M*.) *martiniquensis*		MiSeq fragment length, bp†
	Accession no.	ID, %		Accession no.	ID, %		Accession no.	ID, %		Accession no.	ID, %		Accession no.	ID, %	
ACT	AY079087	97.26		GQ246778	96.86		GQ246777	97.29		ATAF02000030	88.95		NA	–	1,145

ASAT	AM117188	96.21		XM001468861	96.05		XM003879030	97.18		ATAF02000620	86.10		NA	–	1,239
G6PDH	DQ449780	94.49		DQ449784	94.49		XM003878542	95.32		ATAF02000590	83.87		NA	–	1,689
GAPDH	XM003865315	94.88		XM001469791	94.98		XM003874537	96.38		ATAF02000651	85.61		KX790724	92.48	996
GPI	AM117193	96.42		AM157721	96.42		XM003873109	96.37		ATAF02000109	86.45		NA	–	1,818
ICD	XM003858822	97.17		XM001463643	97.17		XM003872819	96.71		ATAF02000077	88.60		NA	–	1,308
ME	DQ449713	97.10		KU175195	97.10		XM003875841	97.36		ATAF02000294	85.76		NA	–	1,516
MPI	XM003863534	95.66		XM001467805	95.81		AJ300464	96.18		ATAF02000537	81.98		NA	–	1,266
PGD	AM157153	94.31		XM001469106	94.38		XM003879281	95.56		ATAF02000632	84.62		NA	–	1,440
PGM	XM003860539	96.72		XM001465338	96.84		XM003875254	96.33		ATAF02000257	84.50		NA	–	1,770
HSP70	CP022650	95.49		CR812655	95.49		FR799587	96.28		ATAF02000637	87.51		KX790754	93.78	2,175
18S rRNA	GQ332356	99.77		GQ332359	99.77		GQ332360	99.95		ATAF02000030	99.8		AF303938	99.38	2,189
ITS rRNA	FJ753386	87.88		GU045592	88.30		FJ948436	84.65		NA	NA		MK603826‡	99.13	954

*Accession numbers are for GenBank. ID, % indicates percent identity between sequences. ACT, β-actin; ASAT, aspartate aminotransferase; GAPDH, glyceraldeyde-3-phosphate dehydrogenase; GPI, glucose-6-phosphate isomerase; G6PDH, glucose-6-phosphate dehydrogenase; HSP70, heat-shock protein 70; ICD, isocitrate dehydrogenase; ID, identity; ITS, internal transcribed spacer; ME, malic enzyme; MPI, mannose-6-phosphate isomerase; NA, not available; NADP, nicotinamide adenine dinucleotide phosphate; ORF, open reading frame; PGD, PGM, 6-phosphogluconate dehydrogenase; PGM, phosphoglucomutase; –, not applicable. †GenBank accession numbers for the Arizona isolate were MT786532 (ACT), MT786533 (ASAT), MT786534 (G6PDH), MT786535 (GAPDH), MT786536 (GPI), MT786537 (ICD), MT786538 (ME), MT786539 (MPI), MT786540 (PGD), MT786541 (PGM), MT813027 (HSP70), MT776522 (18S rRNA) and ITS-1 rRNA region MT791386 (rRNA-ITS region). Sequences for ME and HSP70 were shorter by 206 and 20–30 bp, respectively, when compared with sequences in GenBank. ‡Only a 37-bp partial sequence of ITS rRNA of *L. (M*.) *martiniquensis* is available for comparison.

## Conclusions

*Leishmania* species associated with human clinical cases are typically prevalent in tropical and subtropical foci and classified into 2 subgenera: *Viannia* and *Leishmania*. Nonetheless, environmental changes might contribute to expansion of natural vectors, reservoirs, and emergence of novel *Leishmania* strains and leishmaniasis in nonendemic areas, posing a new and serious challenge to public health (*5*,*6*).

We report an autochthonous case of CL caused by a previously undescribed *Leishmania* parasite in a patient in Arizona. The integrated interpretation of the clinical information, travel history, parasite morphology, CDC species-specific diagnostic test results, and MLSA/phylogenetic analyses suggest that the isolate from Arizona could be a new strain or species within the subgenus *Leishmania*. This isolate is also genetically distinct at the internal transcribed spacer 2 locus from reported isolates for 18 previous cases of leishmaniasis from Arizona, characterized by CDC over the past 10 years, which were detected in travelers returning from disease-endemic areas.

Despite these findings, we realize that classification of this parasite cannot be conclusively determined based solely on genetic evidence observed in this study. Therefore, further investigations (including multilocus enzyme electrophoresis and whole-genome sequencing with next-generation sequencing long read fragments) will be needed to confirm whether the isolate from Arizona is a new species or a new strain in the subgenus *Leishmania*.

Historically, human leishmaniasis in the United States has been considered an exotic, travel-acquired infection. However, this concept must be reexamined because of the expansion of sylvatic animal reservoirs and natural sand fly vectors of *Leishmania* spp. and reports of human and animal autochthonous cases in several states (*7*–*9*,*13*–*15*). Considering the patient’s travel history, the increased reports of zoonotic cases, and the active presence of sand fly vectors/reservoirs in southern areas of the United States, we concluded that the CL reported was probably caused by local parasite transmission. Because there is increasing evidence of likely local transmission, leishmaniasis could be emerging in the southwestern United States.
